# Direct cyanation reaction from benzoic acid to benzonitrile by paired electrosynthesis in liquid ammonia†

**DOI:** 10.1039/d5ra01378j

**Published:** 2025-05-27

**Authors:** Yuki Maeda, Kiyoshi Sakuragi, Makoto Kawase

**Affiliations:** a Energy Chemistry Division, Energy Transformation Research Laboratory, Central Research Institute of Electric Power Industry Yokosuka 240-0196 Japan maeda3937@criepi.denken.or.jp

## Abstract

Nitriles are essential intermediates in organic synthesis processes and are widely used in various industries. Several nitrile synthesis methods have been reported. Among these, the cyanation of carboxylic acids, which are abundant in nature, has attracted significant attention for the valorisation of biomass-derived components. However, these reactions require expensive catalysts, toxic reagents, and high-temperature/-pressure conditions. Herein, we propose a novel cyanation reaction of benzoic acid to benzonitrile, which is achieved by electrolysis in liquid ammonia at room temperature. In this reaction, benzoic acid is reduced to benzyl alcohol, and the iodide anion derived from the supporting electrolyte is oxidised to iodine. Following the electrochemical reactions, benzyl alcohol and iodine react chemically in liquid ammonia to form benzonitrile. The reaction is a paired electrosynthesis process because the products generated on the cathode (benzyl alcohol) and anode (iodine) react to form the final product (benzonitrile). The current efficiency of the electrochemical reduction of benzoic acid to benzyl alcohol and the conversion rate from benzyl alcohol to benzonitrile were 32% and 6% after 1 h of electrolysis, respectively. We also observed that the Pb cathode becomes porous during electrolysis, which facilitates the electrochemical reduction of benzoic acid. This novel reaction enables direct nitrile synthesis from carboxylic acids at room temperature without the use of toxic reagents or expensive catalysts. These findings confirm that the proposed reaction is a novel green cyanation method for carboxylic acids and provides new insights into electrochemical reactions in liquid ammonia for organic synthesis.

## Abstract 2

Electrolysis in liquid ammonia achieved a direct conversion from benzoic acid to benzonitrile by paired electrosynthesis.

## Graphical abstract

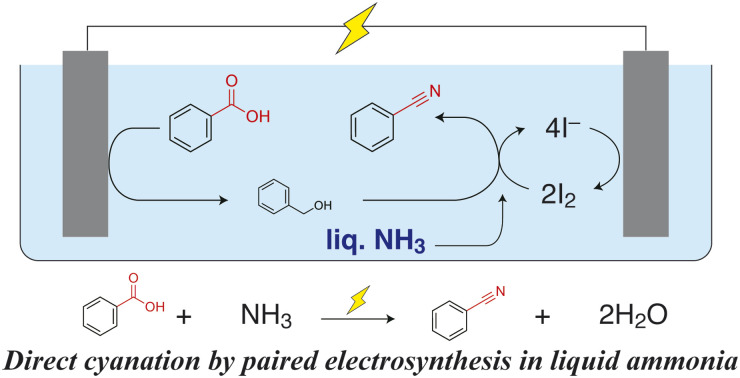

## 1 Introduction

Nitriles are important organic compounds that are commonly used in chemical products^1^ and pharmaceuticals.^2^ In addition, nitriles are useful intermediates in organic syntheses because of their facile conversion into other functional groups and nitrogen-containing heterocyclic compounds.^3,4^ Benzonitrile, the simplest aromatic nitrile, is an industrially valuable chemical that is widely used as a solvent, ligand, intermediate, and precursor.

To produce benzonitrile, the cyanation reactions of benzene halides,^5,6^ benzaldehyde,^7,8^ and benzoic acid^7,9,10^ have been reported. In particular, the cyanation of carboxylic acids, which are abundant in nature, has attracted attention for the green synthesis of nitriles and the valorisation of biomass-derived materials. Despite the advantages of benzoic acid cyanation, these reactions require a significant number of chemicals, high-temperature and high-pressure (350 °C, 65 bar) conditions, and long reaction times. Therefore, the direct synthesis of benzonitriles from benzoic acids under mild conditions is highly desirable.

Ammonia is a candidate nitrogen source for cyanide-free cyanation reactions, such as ammoxidation.^11,12^ Ammoxidation is used to produce benzonitrile from using gaseous ammonia as the nitrogen source. However, ammoxidation requires a harsh environment and expensive catalysts and cannot be applied to the cyanation of carboxylic acids. Thus, alternative approaches are required for the cyanation of carboxylic acids with ammonia.

To transform benzoic acid into benzonitrile, we focused on electrochemical reactions in liquid ammonia, which can effectively dissolve organic and inorganic materials.^13^ Since ammonia is liquified by cooling to −33 °C under ambient pressure or pressurising to 0.85 MPa at room temperature, the solvent is easily separated from the products by evaporation under ambient pressure and temperature. In addition to the advantage of liquid ammonia as a solvent, we focused on the fact that benzoic acid was electrochemically reacted in various solvents to benzyl alcohol,^14^ 4-hydroxybenzoic acid,^15^ and radical intermediates.^16^ Under effective electrochemical conditions in liquid ammonia, a reaction between benzoic acid and ammonia is expected, although this has not been reported to date.

In this study, we investigated the electrochemical reactions of benzoic acid in liquid ammonia (as the solvent and nitrogen source for cyanation) and developed a novel electrochemical cyanation reaction to convert benzoic acid to benzonitrile. This reaction enables the direct production of benzonitrile from benzoic acid *via* electrosynthesis in liquid ammonia at room temperature. Compared to other reported cyanation reactions, the proposed paired electrosynthesis reaction in liquid ammonia proceeds in one step under mild conditions (ambient temperature and a moderate pressure of 0.85 MPa) with a short reaction time, thereby eliminating the need for toxic reagents such as cyanide.

## 2 Results and discussion

### 2.1 Electrolysis of benzoic acid in liquid ammonia

**Fig. 1:**
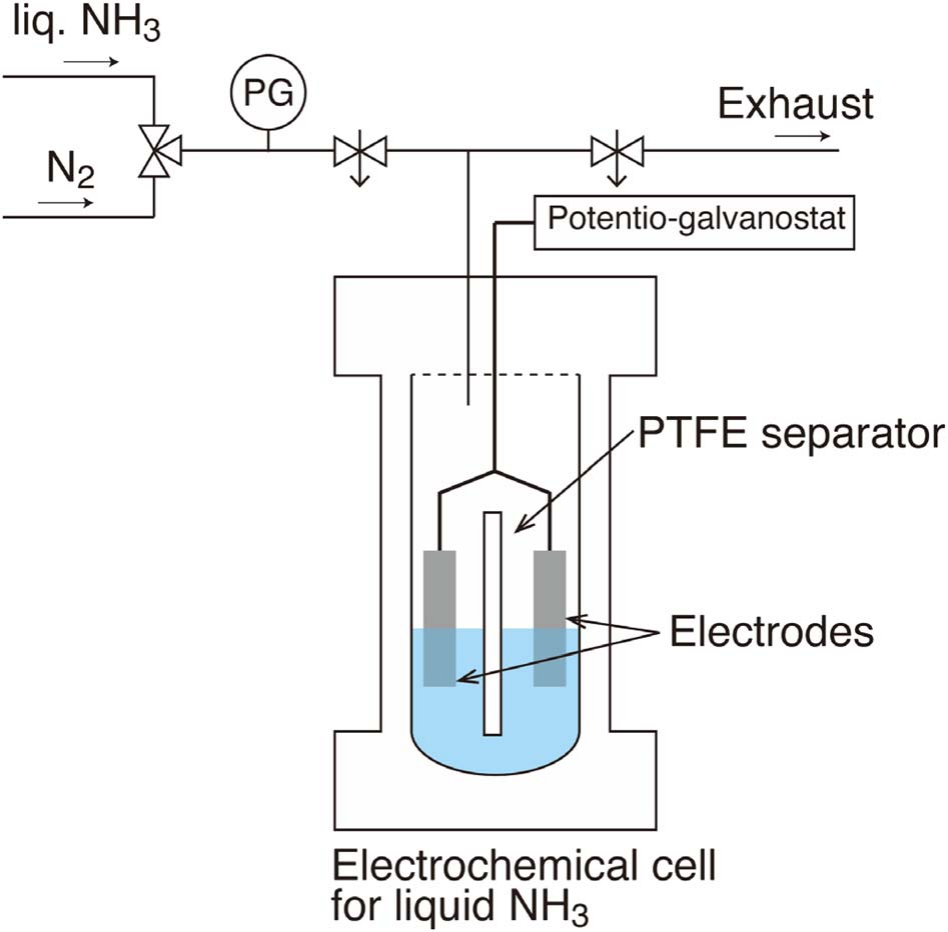
Schematic of the pressure-resistant electrochemical cell for liquid ammonia. The anode and cathode were divided by a PTFE separator. Electrolysis was conducted in the cell at 20 °C and 0.85 MPa without stirring. PG: pressure gauge.

A pressure-resistant electrochemical cell was developed to enable the use of liquid ammonia as a solvent at room temperature, as illustrated in Fig. 1. The electrolyte solution contained 0.1 M (mol dm^−3^) benzoic acid and 0.2 M potassium iodide (KI; supporting electrolyte) in 30 mL of liquid ammonia. Platinum (Pt) mesh was used as both the anode and cathode. For comparison, a similar electrolysis was performed in a 28% aqueous ammonia solution. Constant-current electrolysis was conducted at 50 mA in each electrolyte. The total charge was maintained at 600 C. The products were analysed using gas chromatography–mass spectrometry (GC–MS).

**Fig. 2:**
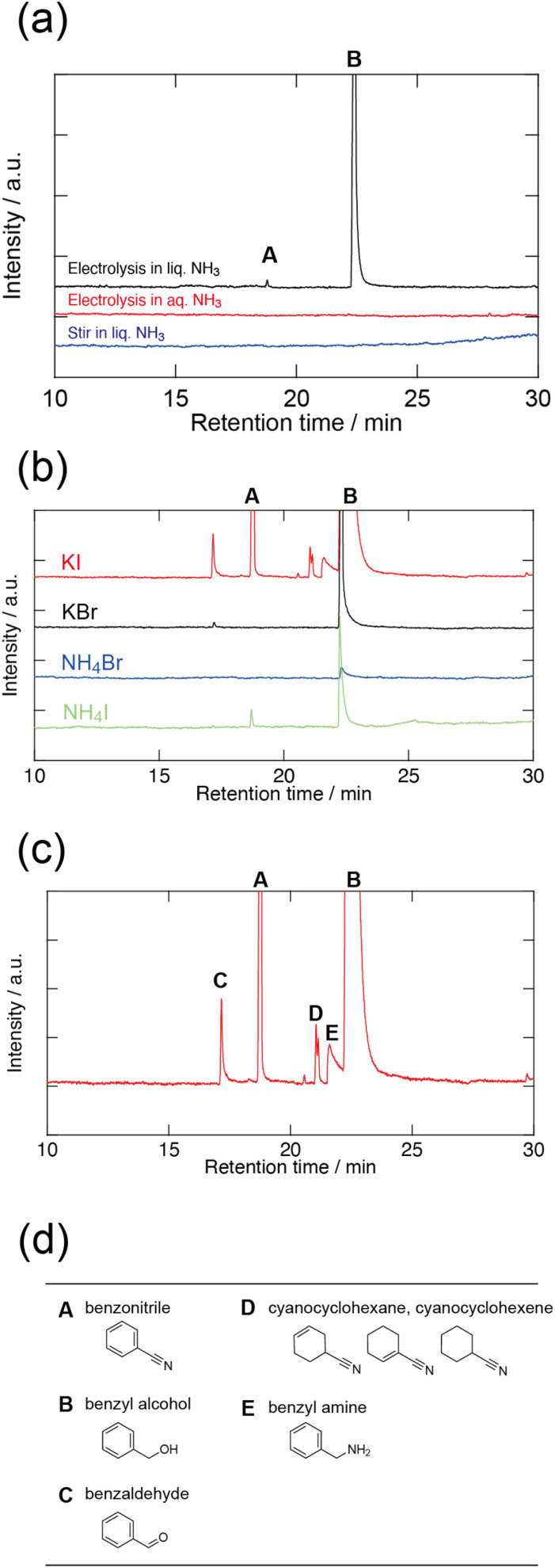
(a) GC–MS chromatograms of the products after the electrolysis of 0.1 M benzoic acid + 0.2 M KI in liquid ammonia (black line) and in aqueous ammonia solution (red line). The blue line was obtained using 0.1 M benzoic acid + 0.1 M KI + 0.1 M I_2_ in liquid ammonia stirred for 3 h without electrolysis. Benzonitrile and benzyl alcohol were detected only in the electrolysis in liquid ammonia. (b) GC–MS chromatograms of the products after the electrolysis of 0.1 M benzoic acid + 0.2 M supporting electrolyte (KI, NH_4_I, KBr, NH_4_Br) in liquid ammonia using a Pb cathode. Although benzyl alcohol was detected in all electrolytes, benzonitrile was detected only in the electrolyte with KI and NH_4_I. (c) Enlarged chromatogram of the red line in (b). (d) Chemicals identified from the chromatograms.

Fig. 2(a) shows the GC–MS chromatograms of the products after electrolysis in liquid and aqueous ammonia. Benzonitrile (A) and benzyl alcohol (B) were synthesised *via* electrolysis of benzoic acid in liquid ammonia. In contrast, no reaction was observed in aqueous ammonia, indicating that the reaction is specific to liquid ammonia. To confirm whether this reaction is indeed the result of electrolysis, 0.1 M benzoic acid, 0.1 M KI, and 0.1 M iodine (I_2_) were stirred in liquid ammonia for 3 h without electrolysis. Note that the colour of the electrolyte around the anode turned yellow during electrolysis, suggesting that iodide ions (I^−^) were electrochemically oxidised at the anode to form I_2_ (Fig. S1(a)†). To confirm the reaction between benzoic acid and I_2_, I_2_ was also added to the electrolyte. Stirring alone did not induce this reaction, indicating that benzoic acid did not react with KI, I_2_, or ammonia without electrolysis. Thus, the electrolysis of benzoic acid in liquid ammonia is required for the cyanation reaction.

Fig. 2 suggests that benzyl alcohol is synthesised *via* the electrochemical reduction of benzoic acid. In liquid ammonia, the self-dissociation of ammonia (2NH_3_ ⇄ NH_4_^+^ + NH_2_^−^, K = [NH_4_^+^][NH_2_^−^] = 10^−33^ at 223 K) and the acid dissociation of benzoic acid occur as shown in eqn (1).

1 C_6_H_5_COOH + NH_3_ ⇄ C_6_H_5_COO^−^ + NH_4_^+^

Thus, the electrochemical reactions at the cathode and anode are presented below.

2 Cathode: C_6_H_5_COO^−^ + 5NH_4_^+^ + 4e^−^ → C_6_H_5_CH_2_OH + H_2_O + 5NH_3_

3 Anode: 2I^−^ → I_2_ + 2e^−^

It has been reported that benzoic acid is reduced to benzyl alcohol *via* electrolysis in a water–ethanol electrolyte mixture, and the current efficiency is effectively increased by using a lead (Pb) cathode.^14^ To increase the current efficiency of the electrochemical reduction of benzoic acid to benzyl alcohol, electrolysis was performed using a Pb cathode. Moreover, the influence of the supporting electrolyte was investigated to obtain insights into the benzonitrile reaction pathway. KI, ammonium iodide (NH_4_I), potassium bromide (KBr), and ammonium bromide (NH_4_Br) were used as supporting electrolytes. The electrolysis was prematurely terminated at 165 C when using KI as the supporting electrolyte, owing to the collapse of the Pb electrode during electrolysis (Fig. S1(b)†). Fig. 2(b) shows the GC–MS chromatograms of the electrolysis products in various supporting electrolytes. The synthesis of benzyl alcohol in the electrolyte was confirmed using all supporting electrolytes. This indicates that the electrochemical reduction of benzoic acid is not dependent on the supporting electrolyte. In contrast, benzonitrile is identified only in the presence of KI and NH_4_I. This indicates that I^−^ and/or I_2_ are necessary for the conversion to benzonitrile. In terms of the reaction with benzyl alcohol and I_2_, previous works have reported that alcohols and aldehydes react with iodine in aqueous ammonia to form nitriles (eqn (4)).^8,17^

4 C_6_H_5_CH_2_OH + 2I_2_ + 5NH_3_ → C_6_H_5_CN + H_2_O + 4NH_4_^+^ + 4I^−^

Thus, at the cathode, benzoic acid is reduced to benzyl alcohol (eqn (2)), while at the anode, I^−^ is oxidised to I_2_ (eqn (3)). The electrochemically produced benzyl alcohol and I_2_ react chemically with ammonia to form benzonitrile in liquid ammonia. In this reaction, I_2_ is chemically regenerated to I^−^. The overall reaction derived from eqn (1)–(4) is as follows:

5 C_6_H_5_COOH + NH_3_ → C_6_H_5_CN + 2H_2_O

**Fig. 3:**
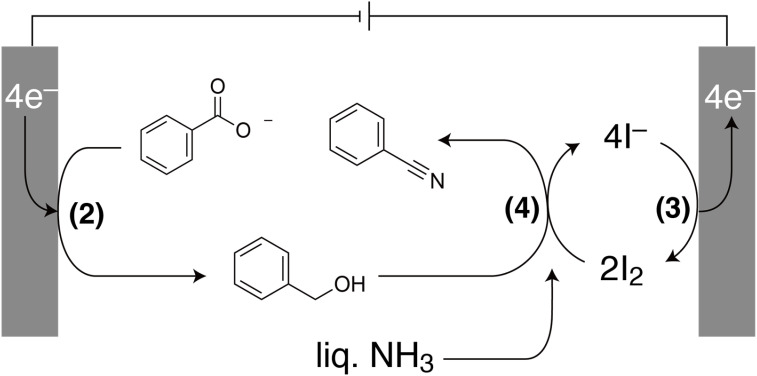
Schematic of the paired electrosynthesis of benzoic acid in liquid ammonia. Benzoic acid is reduced to benzyl alcohol at the cathode (eqn (2)) and I^−^ is oxidized to I_2_ at the anode (eqn (3)). Benzyl alcohol and I_2_ react with ammonia to produce benzonitrile (eqn (4)).

These results indicate that the conversion of benzoic acid to benzonitrile occurs *via* the paired electrosynthesis reaction illustrated in Fig. 3. Eqn (5) suggests that, in addition to benzonitrile, only water and the supporting electrolyte (KI) remain after evaporation of liquid ammonia. Because KI is dissolved in the water but benzonitrile is not, the benzonitrile is easily separated. This is a major advantage of this reaction.

### 2.2 Side reactions of the paired electrosynthesis

**Fig. 4:**
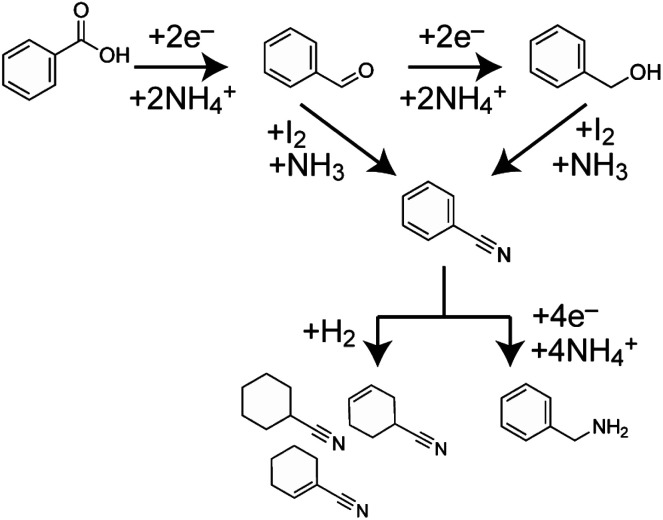
Plausible reaction pathway for the paired electrosynthesis of benzoic acid to benzonitrile in liquid ammonia. The main reactions are the electrochemical reduction of benzoic acid to benzyl alcohol and the chemical conversion from benzyl alcohol to benzonitrile with I_2_ in liquid ammonia. Benzaldehyde is the intermediate of the electrochemical reduction of benzoic acid and is also converted to benzonitrile. During electrolysis, benzonitrile is excessively reduced to cyanocyclohexane and benzylamine.

As shown in Fig. 2(c), some by-products with relatively weak peak intensities were identified. These by-products were benzaldehyde (C), cyanocyclohexane and cyanocyclohexene (D), as well as benzylamine (E). Based on the identified by-products, a plausible reaction of benzoic acid to benzonitrile with side reactions is summarised in Fig. 4. C represents an intermediate in the reduction of benzoic acid to benzyl alcohol. Additionally, benzaldehyde is converted into benzonitrile *via* a reaction between I_2_ and ammonia.^8^ This indicates that the direct conversion of benzaldehyde to benzonitrile is a possible reaction pathway. D is synthesised from benzonitrile, which is reduced by electrochemically generated solvated electrons and H_2_.^18^E can be synthesised from benzonitrile *via* electrochemical reduction.^19^ These results indicate that by-products D and E are compounds resulting from the over-reduction of benzonitrile. To inhibit excessive reduction, flow electrolysis could be used to increase the efficiency of benzonitrile production. Moreover, paired electrosynthesis in liquid ammonia produces benzonitrile and benzylamine. This suggests that the reaction can be applied to the amination of benzoic acid to benzylamine by controlling the electrolysis conditions, including prolonging the reaction time and optimising the applied potential.

### 2.3 Current efficiency and conversion rate of paired electrosynthesis

The current efficiency of benzoic acid to benzyl alcohol conversion was calculated based on the GC–MS results and the total charge. It was assumed that the reduction to benzyl alcohol is a 4-electron reaction (eqn (2)), and that all benzonitrile was produced from benzyl alcohol such that the total amount of the produced benzyl alcohol is equal to the sum of the calculated amount of benzyl alcohol and benzonitrile from GC–MS. Table 1 presents the current efficiency and conversion rate. Note that the current efficiency and conversion rate do not consider the by-products discussed above. Because the by-products were mainly derived from benzonitrile, the actual current efficiency and conversion rate should be larger than those listed in Table 1.

**Table 1:** Current efficiency of the electrochemical reduction of benzoic acid to benzyl alcohol in liquid ammonia and the conversion rate from benzyl alcohol to benzonitrile under constant-current electrolysis of 50 mA

Cathode	Supporting electrolyte	Total charge/C	Equivalents of electron/F mol^−1^	Benzyl alcohol yield/%	Current efficiency/%	Benzonitrile yield/%	Conversion rate/%
Pt	KI	600	0.52	0.36	0.69	0.0052	0.51
Pb	KI	165	0.14	4.5	32	0.28	6.1
Pb	NH_4_I	600	0.52	0.099	0.19	0.0066	6.8
Pb	KBr	600	0.52	0.27	0.51	0	0
Pb	NH_4_Br	600	0.52	0.017	0.033	0	0

Compared with the Pt electrode, the Pb electrode drastically improved the current efficiency, despite the low total charge of 165 C due to the decomposition of the Pb electrode. In the case of the NH_4_I supporting electrolyte, the current efficiency is lower than that obtained using KI. The applied voltages during the constant-current electrolysis of 50 mA are shown in Fig. S2.† The voltage was about 7 V in the electrolyte with KI, KBr, and NH_4_Br. On the other hand, the voltage was about 5 V in the electrolyte with NH_4_I. Although the applied voltages with KI, KBr, and NH_4_Br were the same, the current efficiency of the electrochemical reduction of benzoic acid to benzyl alcohol was extremely high only with KI. This indicates that the current efficiency is independent on the applied voltage. Note that the constant-voltage electrolysis of 7 V in the electrolyte with NH_4_I also resulted in the low current efficiency. This also means that the applied voltage has nothing to do with the current efficiency.

Compared between the electrolyte with NH_4_^+^ and K^+^ salts, the current efficiency in the electrolyte including NH_4_^+^ is lower than that in the electrolyte including K^+^. This suggests that the concentration of NH_4_^+^ influences the current efficiency.

**Fig. 5:**
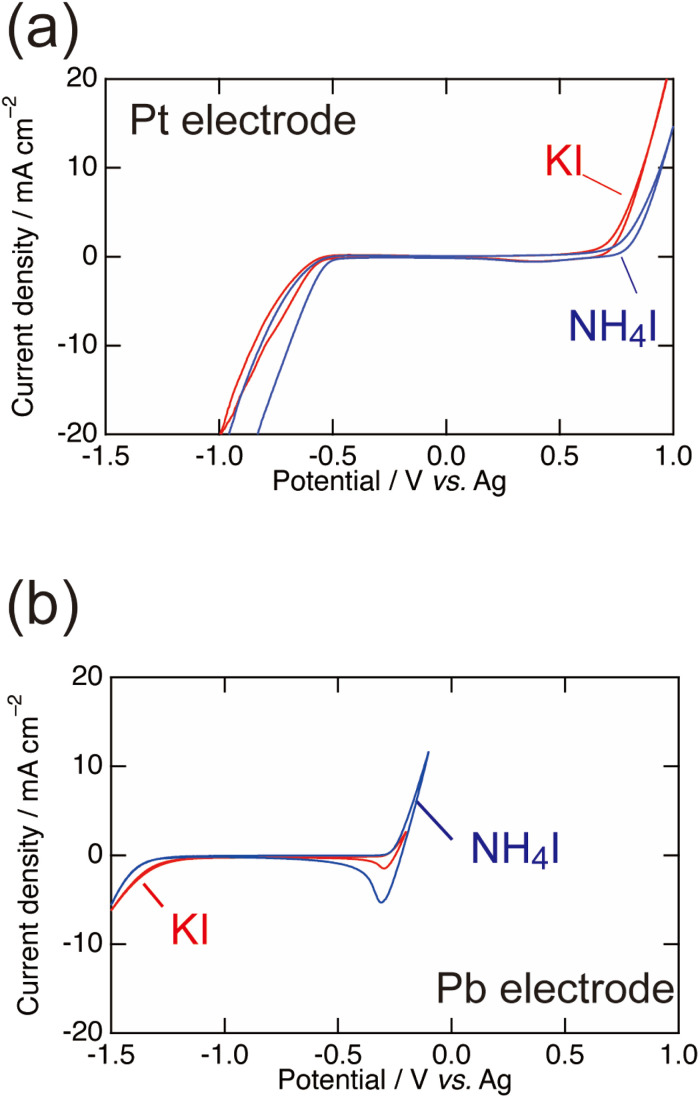
CV curves of 0.1 M benzoic acid in liquid NH_3_ on (a) Pt and (b) Pb electrodes. The supporting electrolytes were 0.2 M KI or NH_4_I and a scan rate of 100 mV s^−1^ was used. There is no difference between KI and NH_4_I. On the Pt electrode, I_2_ production and H_2_ evolution occur. On the Pb electrode, Pb dissolution and deposition occur, and the potential where H_2_ evolution starts is shifted to a lower potential compared with the Pt electrode.

To characterise the electrochemical behaviour when using different electrodes (Pb or Pt) and supporting electrolytes (KI and NH_4_I), cyclic voltammetry (CV) was conducted in 0.1 M benzoic acid + 0.2 M KI or NH_4_I with Pt or Pb electrodes (Fig. 5). Current peaks related to the reduction of benzoic acid were not detected because of the high currents of other electrochemical reactions, such as H_2_ and I_2_ evolution. The I^−^/I_2_ redox reaction (eqn (3)) at the anode was observed at around 0.5 V for Pt, while the electrochemical dissolution and deposition of Pb occurred at around −0.4 V for Pb. This indicates that the Pb electrode is easily dissolved by anodic polarisation in liquid ammonia. In the case of cathodic reactions, a large cathodic current for H_2_ evolution from ammonia is observed (eqn (6)).

6 2NH_3_ + 2e^−^ → H_2_ + 2NH_2_^−^

Fig. 5 shows that H_2_ evolution from ammonia occurs at a more negative potential on Pb than on Pt. The Pb cathode achieves a higher current efficiency than the Pt cathode because Pb has the large overpotential of H_2_ evolution. However, Table 1 indicates that the current efficiency for the Pb electrode in the NH_4_I electrolyte is quite low, although the H_2_ evolution behaviour shown in the CV curve is similar to that observed with the KI electrolyte. Thus, an explanation other than the overpotential of H_2_ evolution is required.

Although there is no difference in the CV curves obtained using KI or NH_4_I, the decomposition of Pb occurs only in the KI electrolyte during the cathodic scan. This suggests that the chemical reaction with Pb following the electrochemical reactions occurs during electrolysis.

It has been reported that Pb hydride is formed by H_2_ evolution, which occurs *via* cathodic polarisation of the Pb electrode.^20^ Pb hydride is unstable and decomposes into Pb nanoparticles. Consequently, the Pb electrode collapses and is dispersed as nanoparticles in the electrolyte, leaving behind a porous Pb electrode. Furthermore, this reaction did not occur when an acid was introduced into the electrolyte. In our system, a robust base of NH_2_^−^ is generated alongside H_2_ evolution in liquid ammonia (eqn (6)), indicating that the electrolyte around the Pb cathode becomes strongly basic during electrolysis. Thus, hydride formation and decomposition occur in the liquid ammonia. When using NH_4_I as the supporting electrolyte, no decomposition occurs because NH_4_^+^, which acts as an acid in liquid ammonia, neutralises NH_2_^−^.

**Fig. 6:**
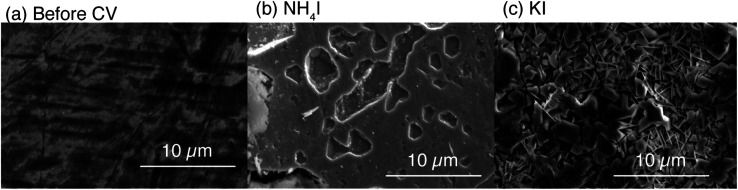
Surface SEM images of Pb electrodes (a) before CV, and after CV in (b) NH_4_I or (c) KI electrolytes. The surface after CV in the NH_4_I electrolyte is still smooth, although the roughness is slightly higher than that of the initial surface due to anodic dissolution. In contrast, a porous Pb surface was formed in the KI electrolyte.

To confirm the Pb decomposition, scanning electron microscopy (SEM) images of the Pb cathode surfaces were obtained after CV scans in KI or NH_4_I electrolytes, as shown in Fig. 6. Fig. 6(a) and (b) show that the cathode surface after CV in the NH_4_I electrolyte is still quite smooth. In contrast, a highly porous surface is observed after the electrolysis in the KI electrolyte (Fig. 6(c)), which is in good agreement with a previous report.^20^ These findings confirm the cathodic decomposition of Pb in a basic solution of liquid ammonia.

A previous study reported that the Pb cathode loses its activity during the electrochemical reduction of benzoic acid after prolonged use, and a clean Pb surface is necessary to achieve a high reduction activity.^14^ Because Pb decomposition reveals a clean surface in the KI electrolyte, the activity of Pb should be maintained. Moreover, porous Pb electrodes formed by decomposition exhibit activity during the electrochemical reduction of oxalic acid.^20^ This suggests that porous Pb also exhibits activity in the electrochemical reduction of benzoic acid, as well as oxalic acids. This clarifies the high current efficiency observed in the electrolyte with KI.

Huang *et al.* reported that the hydride formation also occurred on Sn.^20^ In addition, Swann *et al.* reported that the electrochemical reduction of benzoic acid to benzyl alcohol did not occur on Sn, Hg, Zn, Al, Ni, Cu and Fe electrodes, but occurred on Cd and Pb electrodes in water–ethanol mixed solution.^14^ This indicates that the hydride formation occurs on Sn, but the electrochemical reduction of benzoic acid on Sn does not. This suggests that other effects such as the interaction between benzoic acid and the electrode surface in addition to the hydride formation still exist on the benzoic acid reduction.

To confirm the relationship between the Pb decomposition and current efficiency, the constant-current electrolysis of 50, 30, 10 mA were conducted in the electrolyte with KI using Pb cathode. The total charge was 165 C. The applied voltages are shown in Fig. S3.† The results are summarized in Table 2. In the electrolysis of 10 mA, Pb decomposition was negligible, and benzyl alcohol and benzonitrile were not detected. In that of 30 mA, Pb started to decompose about one hour after the electrolysis begun, and Pb was partially decomposed in the end of the electrolysis. The current efficiency was 6.6%. This result suggests that the redox potential of benzoic acid to benzyl alcohol is more negative than that of Pb decomposition, and/or the Pb decomposition affects the electrochemical reduction of benzoic acid. The detailed investigation on the electrode materials will be conducted in future works.

**Table 2:** Current efficiency of the electrochemical reduction of benzoic acid to benzyl alcohol in liquid ammonia and the conversion rate from benzyl alcohol to benzonitrile under constant-current electrolysis using Pb cathode

Applied current/mA	Decomposition of Pb electrode	Total charge/C	Benzyl alcohol yield/%	Current efficiency/%	Benzonitrile yield/%	Conversion rate/%
50	Yes	165	4.5	32	0.28	6.1
30	Yes	165	0.92	6.6	0.0052	0.55
10	No	165	0	0	0	0

From the above discussion, the Pb decomposition triggers the electrochemical reduction of benzoic acid. The reduction peak potentials of the one-electron reduction from benzoate (C_6_H_5_COO^−^) to the radical anion ((C_6_H_5_C·(O^−^)_2_)) in various ionic liquids have been reported to be from −1 to −3 V *vs.* Ag on Pt or Au electrodes.^16^ This suggests that the reduction of benzoic acid also starts from −1 to −3 V *vs.* Ag in liquid ammonia. The cathodic scan over −2 V, however, cannot be precisely recorded due to the Pb decomposition. This is because the current peak of benzoic acid is not observed in CVs in Fig. 5 even though the high current efficiency of 32% was achieved in the electrolyte with KI on Pb electrode.

**Fig. 7:**
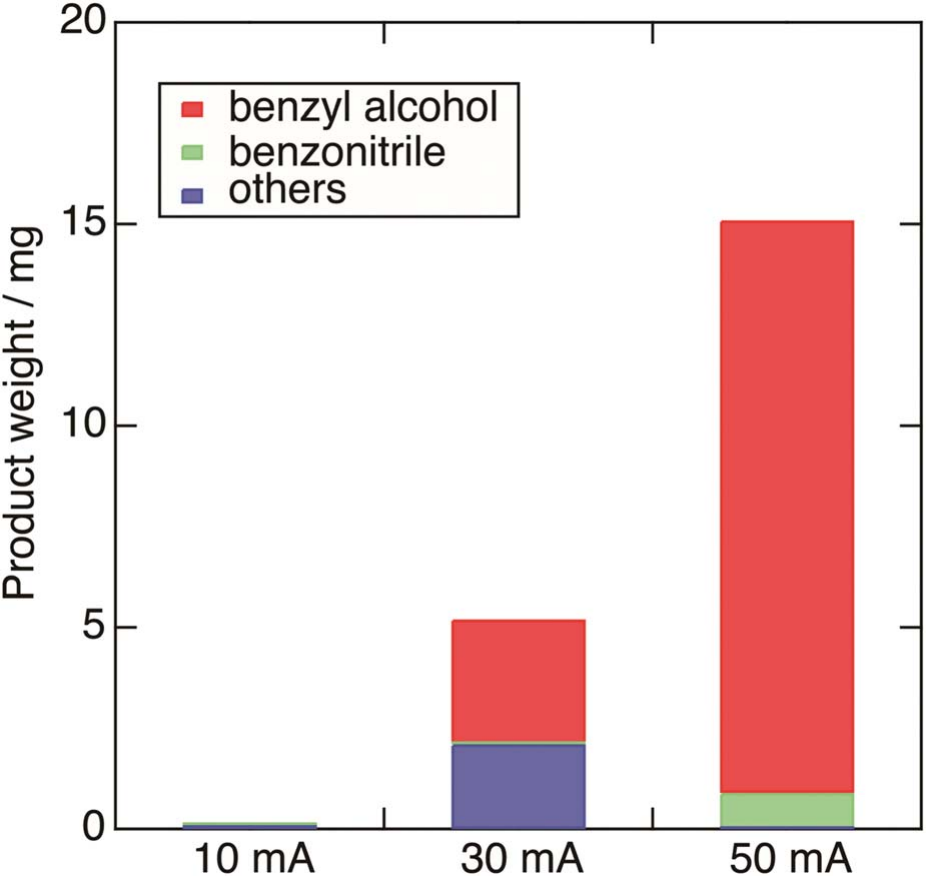
The product weight in the electrolysis of 10, 30, and 50 mA. The weight of benzyl alcohol and benzonitrile were calculated from the results of GC–MS. Others indicate the weight subtracting the weight of benzyl alcohol and benzonitrile from the total weight of the crude.

In addition, the product weight in the electrolysis of 10, 30, and 50 mA are shown in Fig. 7. The weight of benzyl alcohol and benzonitrile were calculated from the results of GC–MS. Others indicate the weight subtracting the weight of benzyl alcohol and benzonitrile from the total weight of the crude. In the electrolysis of 10 mA, the total weight of the products was 0.1 mg. As the current increases, the total weight of the products and the weight ratio of benzyl alcohol and benzonitrile increase.

The conversion rate of benzyl alcohol to benzonitrile was calculated from GC–MS. It was assumed that conversion rate was calculated by dividing the amount of benzonitrile by the total amount of benzyl alcohol from GC–MS. The conversion rate is almost the same regardless of the supporting electrolyte. In a previous study, benzyl alcohol and I_2_ were converted to benzonitrile with a conversion rate of 82% by stirring in aqueous ammonia at 60 °C for 2 h.^17^ However, in our study, when benzyl alcohol (1 mmol) and I_2_ (2 mmol) were stirred in liquid ammonia at room temperature for 2 h, a conversion rate of 13% was obtained. These results indicate that the conversion rate of benzyl alcohol to benzonitrile is slower in liquid ammonia at room temperature than in aqueous ammonia at 60 °C. In addition, I_2_ is consumed in liquid ammonia through the reaction described in eqn (7):^21^

7 3I_2_ + 8NH_3_ → N_2_ + 6NH_4_^+^ + 6I^−^

To efficiently utilise I_2_ for the conversion of benzyl alcohol to benzonitrile (eqn (5)), stirring the electrolyte or flow electrolysis is recommended. The reason for the low conversion rate when using a Pt cathode (0.51%) remains unclear. The effects of stirring, electrolyte temperature, and electrode configuration on the chemical reaction need to be clarified in future work. The optimisation of the electrolysis temperature and electrolyte flow is essential for enhancing the conversion of benzoic acid to benzonitrile.

## 3 Conclusions

We observed that benzoic acid can be directly converted to benzonitrile through paired electrosynthesis in liquid ammonia. During this electrolysis reaction, the reduction of benzoic acid to benzyl alcohol at the cathode and the oxidation of I^−^ to I_2_ at the anode occur. Subsequently, benzyl alcohol and I_2_ react with ammonia to form benzonitrile. The current efficiency of the reduction of benzoic acid to benzyl alcohol is enhanced by employing Pb as the cathode and KI as the supporting electrolyte. This improvement is attributed to the ability of the porous Pb formed by electrolysis to reduce benzoic acid. The conversion rate of benzyl alcohol to benzonitrile in liquid ammonia is lower than that reported previously, where the reaction was stirred in aqueous ammonia at 60 °C. This was due to the consumption of I_2_ in liquid ammonia and the low reaction temperature used in our study. Further improvements are expected by controlling the electrolyte and reaction temperature and applying flow electrolysis.

Typically, the conversion of carboxylic acids such as benzoic acid to nitriles is a multistep reaction that requires toxic reagents such as cyanides, high-temperature and high-pressure environments, and expensive catalysts. Compared to these methods, the proposed reaction offers the distinct advantage of the direct conversion to nitriles through electrolysis in liquid ammonia at room temperature. This method represents a novel green cyanation reaction and is expected to be applicable to various organic compounds containing carboxylic groups.

## 4 Experimental

### 4.1 Electrochemical measurements

All the reagents were extra pure reagents and purchased from FUJIFILM Wako Pure Chemical. The electrodes were welded to polytetrafluoroethylene (PTFE)-coated wires and connected to a potentiostat-galvanostat (Bio-Logic, SP-50), as shown in Fig. 1. After purging the electrochemical cell with N_2_, it was filled with liquid ammonia from a cylinder. Then, the electrolyte was stirred for a few minutes to dissolve the benzoic acid and the supporting electrolyte. The anode and cathode were separated using a PTFE separator. Electrolysis was performed in the cell at 20 °C and 0.85 MPa without stirring. The cathodes were either a Pt mesh electrode (Nilaco, 99.95%) or a Pb electrode (Nilaco, 99.9%). The anode was a Pt mesh electrode. The electrodes were cut into 2 cm × 1 cm pieces and positioned such that the immersed electrode area was 2 cm^2^. In the case of CV, a Pt disk electrode (*φ* = 5 mm), a Pt mesh electrode, and a silver wire (Nilaco, *φ* = 0.5 mm) were used as the working electrode, counter electrode, and quasi-reference electrode, respectively. The electrodes were sonicated in ultrapure water (Milli-Q, Merck) and ethanol before testing. After the CV tests, the electrode surfaces were investigated using field-emission SEM (Hitachi High-Tech, S-4300SE/N).

### 4.2 Analysis of the electrolysis products

Following electrolysis, the cells were gradually opened to the atmosphere, allowing the ammonia to evaporate overnight. To remove the I_2_ generated by electrolysis, 5 mL of saturated sodium sulfite solution was added to the cell, after which the solution was extracted three times with 10 mL of ethyl acetate. The ethyl acetate phase was washed with brine and subsequently dehydrated with magnesium sulfate. Following the evaporation of ethyl acetate, the products were dissolved in 1 mL of ethyl acetate and analysed by GC–MS (Shimadzu, GCMS-QP2010 SE) with a DB-5MS column (30 m × 0.25 mm × 0.25 μm). Because of the residual ammonia and added sodium sulfite, the remaining aqueous phase was a basic solution, meaning that acidic substances such as benzoic acid were dissolved in the aqueous phase.

## Data availability

The data supporting this article have been included as a part of the ESI.†

## Author contributions

Yuki Maeda: conceptualisation, data curation, formal analysis, funding acquisition, investigation, methodology, validation, visualisation, writing the original draft, and project administration. Kiyoshi Sakuragi: Formal analysis, funding acquisition, validation, visualisation, writing – review & editing. Makoto Kawase: Funding acquisition, supervision, visualization, writing – review & editing.

## Conflicts of interest

There are no conflicts to declare.

## Supplementary Material

RA-015-D5RA01378J-s001
