# Supplementary material for: Direct cyanation reaction from benzoic acid to benzonitrile by paired electrosynthesis in liquid ammonia
Source: RSC Adv. 2025 May 27;15(22):17562–9. doi: 10.1039/d5ra01378j (PMC12107531; doi:10.1039/d5ra01378j)
Supplement: RA-015-D5RA01378J-s001 [file RA-015-D5RA01378J-s001.pdf]

Supplementary Information (SI) for RSC Advances.
This journal is © The Royal Society of Chemistry 2025

Supplementary Information

Direct cyanation reaction from benzoic acid to benzonitrile achieved by paired electrosynthesis in liquid ammonia

Yuki Maeda[1,*], Kiyoshi Sakuragi[1], Makoto Kawase[1]

[1]Energy Chemistry Division, Energy Transformation Research Laboratory, Central Research Institute of Electric Power Industry, Yokosuka, 240-0196, Japan

*Corresponding author:

Yuki Maeda; maeda3937@criepi.denken.or.jp

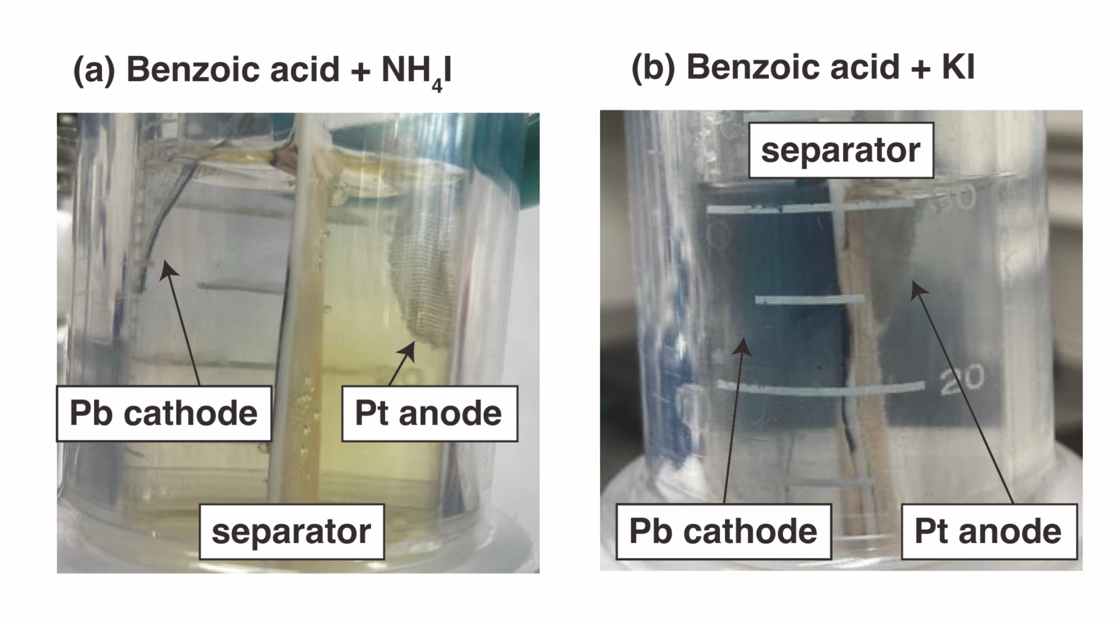

Figure S1 The photographs of the electrochemical cell during the electrolysis in (a) benzoic acid + NH_4I and (b) benzoic acid + KI.

Figure S1(a) shows the color change to yellow at Pt anode, which suggests that I^- is electrochemically oxidized to I_2. In the electrolyte of benzoic acid and KI, Pb is dispersed into the electrolyte during the electrolysis. This decomposition of Pb is negligible in the electrolyte of benzoic acid and NH_4I.

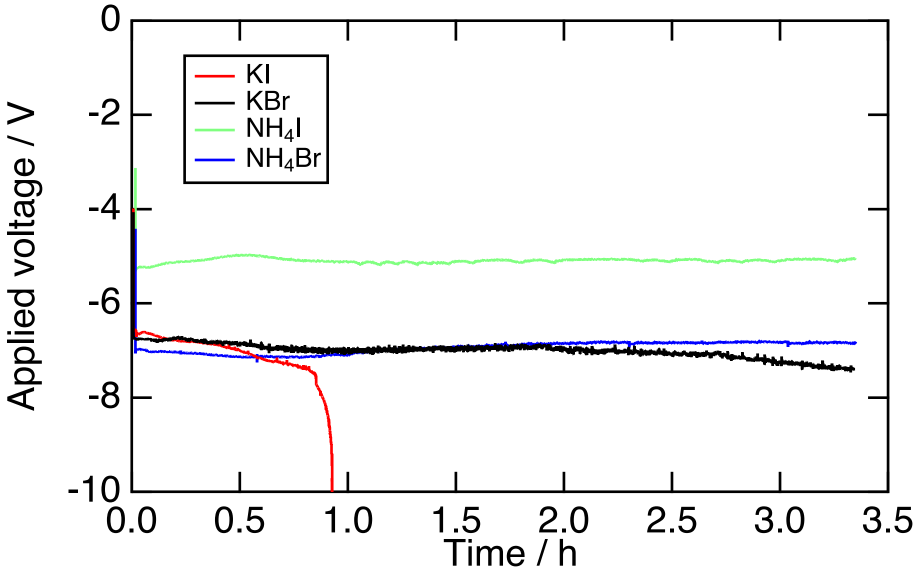

Figure S2 Time dependence of the applied voltage during the constant-current electrolysis of 50 mA in the electrolysis with KI, KBr, NH_4I, NH_4Br. The anode and cathode are Pt mesh and Pb plate, respectively. The applied voltages were stable during the electrolysis. Only in the electrolysis with KI, the electrolysis was abnormally terminated due to the decomposition of Pb electrode.

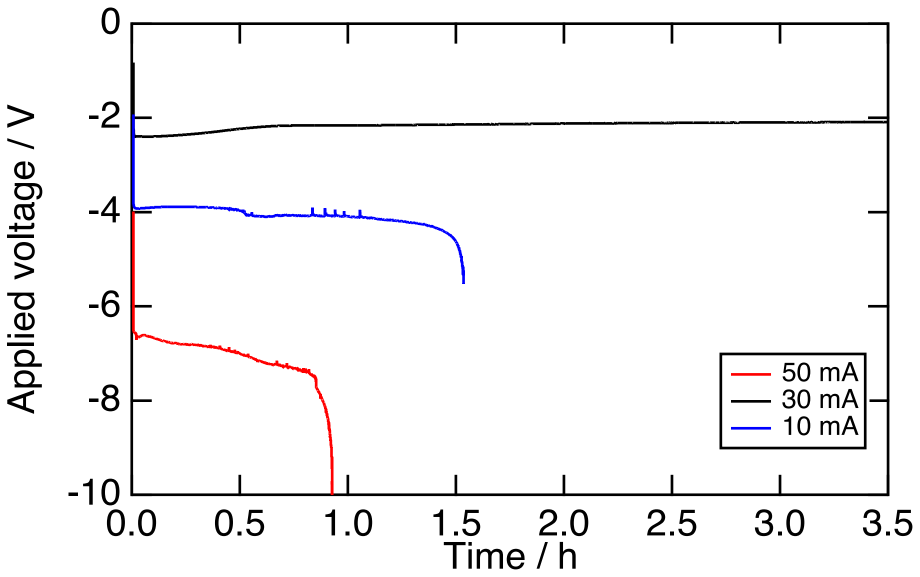

Figure S3 Time dependence of the applied voltage during the constant-current electrolysis of 50, 30, 10 mA. The total charge was 165 C. Pb decomposition occurred in the electrolysis of 50 and 30 mA. In the electrolysis of 50 mA, Pb was completely decomposed, on the other hand, Pb electrode still remained in that of 30 mA.
